# Psychological Profiles and Their Relevance with Temporomandibular Disorder Symptoms in Preorthodontic Patients

**DOI:** 10.1155/2022/1039393

**Published:** 2022-09-30

**Authors:** Chengxinyue Ye, Xin Xiong, Yuyao Zhang, Dan Pu, Jie Zhang, Shufang Du, Jun Wang

**Affiliations:** Department of Orthodontics, National Clinical Research Center for Oral Diseases, State Key Laboratory of Oral Diseases, West China Hospital of Stomatology, Sichuan University, Chengdu 610041, China

## Abstract

**Background:**

Temporomandibular disorders (TMDs) refer to a group of heterogenous musculoskeletal diseases with diverse clinical symptoms and an undetermined aetiology. The psychological profiles were closely related to the onset and treatment outcomes of TMDs.

**Objective:**

To examine the relevance between psychological profiles and different symptoms of TMDs in preorthodontic patients.

**Methods:**

The study was conducted among 570 preorthodontic patients. TMDs symptoms were recorded by the Diagnostic Criteria for TMD (DC/TMD) symptom questionnaire. The seven-item Generalized Anxiety Disorder Scale (GAD-7), the nine-item Patient Health Questionnaire (PHQ-9), and the Pain Catastrophizing Scale (PCS) were used for the evaluation of anxiety, depression, and pain catastrophizing levels. The relevance of three psychological profiles with TMDs and subtypes was evaluated with Spearman's rank correlation test and logistic regression analysis (*P* < 0.05).

**Results:**

34.56% of the enrolled preorthodontic patients were diagnosed with TMDs. Scores of GAD-7, PHQ-9, and PCS were significantly higher in the TMDs group than in the non-TMDs group. Participants with anxiety, depression, or high pain catastrophizing had a higher prevalence of both pain-related TMDs symptoms and intra-articular TMDs symptoms. The correlations among pain-related TMDs, intra-articular TMDs, and scores on the psychological scales were significant (*P* < 0.05). The adjusted logistic regression model revealed that anxiety, depression, and high pain catastrophizing were significant risk factors for TMDs with an odds ratio (OR) of 2.196, 1.741, and 1.601, respectively. Depression was associated with higher pain-related TMDs prevalence (OR = 2.136), while anxiety and depression were associated with higher intra-articular TMDs prevalence (OR = 2.341 and 1.473).

**Conclusion:**

Anxiety, depression, and high pain catastrophizing were comorbid psychological conditions of TMDs. Depression was the top risk factor for pain-related TMDs, while anxiety rendered the highest risk for intra-articular TMDs. Inclusion of psychological assessments in preorthodontic evaluation might yield great benefits in TMDs screening.

## 1. Introduction

Temporomandibular disorders (TMDs) refer to a group of heterogeneous musculoskeletal diseases affecting the masticatory muscles, the temporomandibular joint (TMJ), and associated structures [1]. TMDs are the most common cause of orofacial chronic pain [2], varying from TMJ pain, masticatory muscle pain, and headaches in the temple. TMDs also greatly interfere with jaw functions, especially the mouth opening and closing process. According to the distinct symptoms, TMDs can be basically classified as pain-related TMDs and intra-articular TMDs by the Diagnostic Criteria for Temporomandibular Disorders (DC/TMD) [3].

The aetiology of TMDs is complex and yet controversial. Both the conventional Research Diagnostic Criteria for Temporomandibular Disorders (RDC/TMD) [4] and the recent DC/TMD recognized a biopsychosocial model for the conceptualization of TMDs. Evidence shows that psychopathological factors may be involved in the initiation, progression, as well as treatment responses of TMDs [5–7]. Anxiety and depression are two highly emphasized aspects, as higher levels of anxiety and depression have been detected among TMDs patients [8, 9]. The seven-item Generalized Anxiety Disorder Scale (GAD-7) and the nine-item Patient Health Questionnaire (PHQ-9) are routinely used in Axis II diagnostic instruments for the screening of anxiety and depression, respectively. Pain catastrophizing is defined as an exaggerated negative mental set toward a painful experience [10]. The Pain Catastrophizing Scale (PCS), a validated assessment for pain catastrophizing, has been used in predicting pain severity and rehabilitation outcomes in chronic pain patients [11, 12]. In the pathology of TMDs, pain catastrophizing has been discovered to be related to pain persistence and treatment responses [13, 14]. Nevertheless, the links between those psychological profiles and different symptoms of TMDs are still to be demonstrated.

In clinical practice, symptoms of TMDs have become recurring complaints among orthodontic patients [15], and a higher prevalence of TMDs has been observed in orthodontic patients than in the general population [16, 17]. Malocclusion, as well as psychological distress following appearance unsatisfaction, may be potential risk factors for TMDs [18]. Therefore, it is of great value to study TMDs based on preorthodontic patients. This study aimed to understand the correlation of psychological factors with different TMDs subtypes and symptoms by analyzing the psychological profiles of 570 preorthodontic patients.

## 2. Materials and Methods

### 2.1. Study Population

This cross-sectional study enrolled 570 consecutive patients who sought orthodontic treatment between September 1, 2021 and March 30, 2022. Written informed consent was acquired from all the participants. The study was approved by the West China Hospital of Stomatology Ethics Committee under document number WCHSIRB-CT-2021-431 and was carried out in compliance with the Helsinki Declaration. The inclusion criteria were as follows: (1) ≥12 years old; (2) capable of comprehending and completing the questionnaires; and (3) with orthodontic complaints. The exclusion criteria were as follows: (1) with a history of TMDs treatment; (2) with a history of tumor, maxillofacial deformity, trauma, or craniofacial surgeries that affected TMJ; (3) history of drug therapy that might hide TMDs symptoms, such as nonsteroidal anti-inflammatory drugs or pain-relief drugs; and (4) currently with acute illness. Demographic information, including age, sex, education level, and general health was collected by a self-reporting questionnaire.

### 2.2. Temporomandibular Disorders Subtypes and Symptoms

The participants were asked to complete a self-reporting DC/TMD symptom questionnaire, and 5 major TMD symptoms (5Ts) of the DC/TMD were recorded to discriminate the subtypes of TMDs [19]. (1) TMD/facial pain: with pain in the jaw, temple, in the ear, or in front of ear; (2) headaches: with headaches including the temple area; (3) TMJ noises: with joint noise when moving the jaw; (4) closed locking: with jaw lock or catch hindering the mouth-opening process; and (5) open locking: with jaw lock or catch hindering the mouth-closing from the wide-open position. Symptoms described in (1) and (2) were categorized as pain-related TMDs symptoms, in consistency with the pain-related TMDs subtype, which included myalgia, arthralgia, or headache attributed to TMDs. Symptoms described in (3), (4), and (5) were referred to as intra-articular TMDs symptoms, matched by intra-articular TMDs subtype, which included disc displacement with reduction and with intermittent locking, disc displacement without reduction with and without limited opening, degenerative joint disorders, or subluxation.

### 2.3. Psychological Profiles

The psychological profiles of the patients were assessed by validated self-reporting questionnaires in the Chinese version. The GAD-7 scale is a seven-item questionnaire for the screening of anxiety [20]. Items are rated based on the frequency of each symptom during the past two weeks, assigned from 0 (not at all) to 3 (nearly every day). A GAD-7 score is obtained by the sum of the items within the range of 0–21. In this study, the cut-off value for the diagnosis of anxiety was ≥5. The PHQ-9 scale is a nine-item questionnaire for the screening of depression [21]. Items are rated based on the frequency of each symptom during the past two weeks, assigned from 0 (not at all) to 3 (nearly every day). A PHQ-9 score is obtained by the sum of the items within the range of 0–27. In this study, the cut-off value for the diagnosis of depression was ≥5. PCS is a thirteen-item questionnaire evaluating the levels of pain catastrophizing [22]. Items are rated based on the frequency of catastrophizing responses towards pain, assigned from 0 (not at all) to 4 (all the time). A PCS total score is obtained by the sum of the items within the range of 0–52. In this study, as we found the mean PCS score in the studied population was lower than that in pain-suffering patients, the cut-off value for high pain catastrophizing was set at ≥10, which comprised around 20% of the sample.

### 2.4. Statistical Analysis

Statistical analyses were performed with EmpowerStats (http://www.empowerstats.com, X&Y Solutions, Inc., Boston, USA). Quantitative data, including age and psychological assessment scores, were presented in the form of mean ± standard deviation and evaluated by the Mann–Whitney *U* test. Categorial data, consisting of sex, education level, history of systemic diseases, as well as the occurrence of TMDs subtypes and symptoms, were presented by frequency (constituent ratio) and evaluated by the R×C chi-square test. The correlation between pain-related TMDs symptoms, intra-articular TMDs symptoms, and scores of psychological assessments were evaluated with Spearman's rank correlation test. A univariate logistic regression analysis was performed to assess the influence of each psychological profile on TMDs and subtypes. Demographic confounders were adjusted in the multivariate regression model. The adjusted variables include sex (‘female' or ‘male'), age (‘above 18' or ‘below 18'),education level (‘college,' ‘postgraduate or above,' or ‘high school or below'), and general health (‘with a history of systemic diseases' or ‘without a history of systemic diseases'). In general, an *α* level of 0.05 was considered statistically significant.

## 3. Results

### 3.1. Prevalence of TMDs and Psychological Profiles in Preorthodontic Patients

A total of 570 patients, among whom 197 (34.56%) were diagnosed with TMDs, were included in this study (Table 1). The average age was 24.44 ± 8.29 years, and the male-to-female ratio was 1 : 2.3, with no statistical significance between the TMDs and the non-TMDs groups. The TMDs group had a higher educational level (1 : 2.5 : 0.6 for high school or below, college, and postgraduate or above, respectively), compared to the non-TMDs group (1 : 1.5 : 0.3). The prevalence of systemic diseases was around 7%, which showed no statistical significance between the two groups.

Average scores of GAD-7, PHQ-9, and PCS were all below the cut-off value in both groups. Generally, anxiety was diagnosed in 19.82% of the total sample, depression in 18.77%, and high pain catastrophizing in 20.70%. The TMDs group endorsed significantly higher scores in three scales than the non-TMDs groups (GAD-7: 2.98 vs. 1.67, PHQ-9: 2.92 vs. 1.84, and PCS: 6.05 vs. 4.51). Consistently, the prevalence of anxiety, depression, and high pain catastrophizing were higher in the TMDs group compared to the non-TMDs group (anxiety: 28.93% vs. 15.01%, depression: 25.38% vs. 15.28%, and high pain catastrophizing: 26.90% vs. 17.43%).

### 3.2. Prevalence of TMDs Symptoms in Different Psychological Profiles

Tables 2–4, present the prevalence of TMDs and symptoms in terms of different psychological profiles. The anxiety group endorsed a higher prevalence of intra-articular TMDs (*P* < 0.01) and all the related symptoms (*P* < 0.05) (Table 2). In pain-related symptoms, only TMD/facial pain displayed statistical significance (*P* = 0.038). Although the prevalence of headaches also increased, no statistical significance was shown (*P* = 0.144).

The prevalence of pain-related TMDs and intra-articular TMDs were significantly higher in the depression group than in the nondepression group (*P* = 0.004 and 0.005 respectively) (Table 3). All five major symptoms appeared at a higher frequency in the depression group with statistical significance (*P* < 0.05).

The high pain catastrophizing group had a larger proportion of those diagnosed with pain-related TMDs (*P*=0.008) and intra-articular TMDs (*P*=0.030) (Table 4). In pain-related TMDs symptoms, a significant difference was shown in headaches (*P*=0.011) but not in TMD/facial pain (*P*=0.128). Among intra-articular TMDs symptoms, TMJ noises and open locking constituted a larger proportion in the high pain catastrophizing group (*P*=0.032 and 0.033, respectively), while closed locking appeared to have no significant difference in prevalence (*P*=0.989).

### 3.3. Correlations between Psychological Profiles and TMDs Symptoms

Spearman's rank correlation revealed the significant correlation between psychological profiles and TMDs symptoms (Table 5). The two subtypes, pain-related TMDs and intra-articular TMDs were positively correlated with each other with a Spearman's correlation coefficient (*r*_s_) of 0.268 (*P* < 0.01). A strong correlation was shown amongst the scores of the three psychological scales (*r*_s_ > 0.5, *P* < 0.01). The correlations between pain-related TMDs and scores of GAD-7, PHQ-9, and PCS were significant (*r*_s_ = 0.121, 0.098, and 0.118, respectively; *P* < 0.05). Intra-articular TMDs were significantly correlated with scores of GAD-7 and PHQ-9 (*r*_s_ = 0.171 and 0.157, *P* < 0.05), but not scores of PCS (*P* > 0.05).

Univariate logistic regression analysis confirmed the influence of the three psychological profiles on TMDs and subtypes (Table 6), and such influence remained after adjustment for demographic confounders (Table 7). All of the three studied psychological profiles, anxiety, depression, and high pain catastrophizing, remained statistically significant in predicting TMDs after adjustment (*P* < 0.05). People with anxiety had 2.196 times the odds of TMDs compared with those without anxiety. The odds ratio (OR) was 1.741 for those with depression and 1.601 for those with high pain catastrophizing. In terms of pain-related TMDs, depression and high pain catastrophizing were associated with a higher prevalence before adjustment, while only depression was distinguished after adjustment (OR = 2.136). For intra-articular TMDs, all three psychological profiles were associated with higher prevalence before adjustment, while anxiety and depression remained statistically significant after adjustment (OR = 2.341 and 1.726, respectively).

## 4. Discussion

Our findings revealed the relevance between psychological profiles and different TMDs subtypes and symptoms. All three psychological factors covered in the study were correlated with TMDs prevalence and clinical symptoms to different extents.

In this study, five major symptoms extracted from the DC/TMD symptom questionnaire were especially focused on and considered the main diagnostic criteria for TMDs and subtypes. Fu et al. affirmed the viability of the five major TMD symptoms (5Ts) as highly sensitive and specific screeners of TMDs and subtypes, with areas under the Receiver Operating Characteristics curve (AUC) as high as 0.98, 1.00, and 0.98 for all TMDs, pain-related TMDs, and intra-articular TMDs [19]. Compared with the internationally accepted DC/TMD diagnostic process [3], clinical and radiographic examinations were not covered in this method, which rendered greater convenience and economic practicability in large-scale population screening. The main defects in applying this screener are the lack of discrimination in taxonomic classifications of TMDs and the lack of information on the duration, severity, and frequency of the symptoms.

To date, the aetiology of TMDs is still considered complex and undetermined. The current consensus is a biopsychosocial model for TMDs [23], recognizing the aggregative role of psychological and social factors in TMDs pathology. As reported in our study and previous literature [9, 24, 25], psychological state and education level (as a social factor) are distributed differently in the TMDs and non-TMDs groups. The DC/TMD included GAD-7 and PHQ-9 as psychological screening tests for clinical and research uses. Pain catastrophizing is a less studied but meaningful psychological indicator. Several studies focused on pain catastrophizing in TMDs, and their results suggested TMDs patients were accompanied by higher pain catastrophizing levels [13, 14], which accorded with our findings. It was hypothesized that the addition of pain catastrophizing might improve the completeness of the diagnosing instruments, especially in pain-related cases [26]. The average score of PCS in our study was lower than that in previous studies on chronic pain [27, 28]. Lack of experimental pain testing and a younger study sample who had fewer intense pain experiences might be the causes. The mechanism of how psychological distress acts in the onset and progression of TMDs is an intriguing issue. It was assumed that neuroendocrine changes, an increase in masticatory muscle tension, and maladaptive oral parafunction in response to psychological distress might be possible mechanisms [29–31]. On the other hand, TMDs commodities such as diffuse pain, impaired oral function, and sleep disturbances might also amplify psychological distress [32, 33].

Since TMDs are heterogenous diseases with diverse clinical symptoms, it is necessary to target different subtypes. The relations between psychological profiles and specific symptoms of TMDs are yet uncertain. Interestingly, our study affirmed the correlation of psychological distress with both pain-related TMDs symptoms and intra-articular TMDs symptoms, which indicated the broad influence of psychological distress. As reported previously [34], pain was the most common reason for TMDs patients seeking TMDs treatment. The pain might come from inflammatory pain of the TMJ, spastic pain of masticatory muscles, somatization of mental stress, or a combination. Dysregulation of the hypothalamic-pituitary-adrenal (HPA) axis caused by anxiety, depression, or stress might be a reasonable explanation for somatic chronic pain [35]. A higher pain catastrophizing level in TMDs patients also implied that the pain in some TMDs patients might be sensitively perceived and responded to in an amplified way. Intra-articular symptoms are commonly seen in disc displacement and degenerative joint diseases, usually indicating dysfunctional or structural disorders of the TMJ. Aberrant and long-term mechanical loading of the TMJ were proposed as the major initiator of intra-articular TMDs [36, 37]. Abnormal psychological status might render changes in muscular tension, leading to disk displacement during jaw movement [38]. Oral parafunction such as grinding or clenching might also increase in frequency, aggravating the overload of the TMJ [39].

Another highlighted finding of this study was the subtle difference between the psychological risk factors for pain-related TMDs and intra-articular TMDs. In the adjusted logistic regression model, depression was the top risk factor for pain-related TMDs, and anxiety was the top risk factor for intra-articular TMDs. The bidirectional relationship between depression and pain was well-established and supported by both biological and cognitive-behavioral views [40, 41]. Some brain regions and neurotransmitters such as serotonin, norepinephrine, and glutamate, were shared in the processing of depression and physical pain [42, 43]. It was reported that antidepressants could have pain-relief effects and were recommended for some pain conditions [44]. Psychologically, depression was frequently accompanied by avoidance, low self-efficacy, and catastrophizing, which could influence the way people appraise pain conditions [45, 46]. The influence of anxiety on intra-articular TMDs was undetermined. One widely accepted explanation was that anxiety might be accompanied by clenching or grinding behaviors that exacerbate the masticatory muscle tension [18, 47]. Pain catastrophizing was assumed as a significant risk factor for TMDs and both subtypes according to univariate analysis, but its association with TMDs subtypes was not statistically significant after adjustment of demographic confounders. It was supposed that the perception of pain might be influenced by some demographic-social factors such as education level [48, 49]. Therefore, the conclusion might differ in studies based on different cultural backgrounds. Some studies suggested that psychological intervention might be beneficial to TMDs treatment [50]. However, evidence were still insufficient to support its application in individualized TMDs treatment, and further studies were greatly anticipated [51].

The study was conducted based on a preorthodontic population and included those who did not come with a TMDs complaint initially. Our results revealed that 1/3 of the participants reported TMDs symptoms. Many studies reported the relation between malocclusion and TMDs, especially intra-articular TMDs [18, 52, 53], although the issue remained controversial [54, 55]. Abnormal psychological profiles have also become more common in preorthodontic patients, probably owing to the increasing appearance anxiety in the new media era and social pressure in youth life stages [56, 57]. In addition to TMDs screening, psychological profiles were also related to orthodontic pain tolerance, treatment compliance, and oral hygiene maintenance in orthodontic patients [58, 59]. Therefore, a better understanding of the psychological aspects of preorthodontic patients might bring multiple benefits in predicting potential problems during treatment.

The primary limitation of this study was the cross-sectional design. Causal relationships between psychological profiles and different TMDs symptoms were undetermined and required a prospective study design. Second, as the psychological scales used were screeners of psychological distress, further diagnosis should be made after consultation with psychiatric or psychological professionals. Last, children under 12 were excluded from the study as they were incapable of completing the self-reporting questionnaires. As previous studies indicated the lower prevalence of TMDs in preadolescents [60], the overall TMDs prevalence from the current study might be overestimated.

## 5. Conclusions

Anxiety, depression, and high pain catastrophizing were comorbid psychological conditions of TMDs. The prevalence of pain-related TMDs symptoms and intra-articular TMDs symptoms were higher in patients with three focused psychological profiles. All three studied psychological profiles were significant risk factors for TMDs. Depression was the top risk factor for pain-related TMDs by raising the risk to 2.136 times, while anxiety rendered the highest risk for intra-articular TMDs by increasing the risk to 2.341 times. For preorthodontic patients, the inclusion of psychological assessments in preorthodontic evaluation might yield great benefits in TMDs screening.

## Figures and Tables

**Table 1:** Patient characteristics in non-TMDs and TMDs groups.

		Overall (*N* = 570)	Non-TMDs (*N* = 373)	TMDs (*N* = 197)	*P* value
*Demography*					
Average age		24.44 ± 8.29	24.23 ± 9.02	24.84 ± 6.70	0.094
Sex	Female	399 (70.00%)	256 (68.63%)	143 (72.59%)	0.338
	Male	171 (30.00%)	117 (31.37%)	54 (27.41%)	
Education level	High school or below	180 (31.58%)	131 (35.12%)	49 (24.87%)	0.041^*∗*^
	College	322 (56.49%)	201 (53.89%)	121 (61.42%)	
	Postgraduate or above	68 (11.93%)	41 (10.99%)	27 (13.71%)	
History of systemic diseases		41 (7.19%)	23 (6.17%)	18 (9.14%)	0.232

*Psychological assessment*					
GAD-7 score		2.12 ± 3.16	1.67 ± 2.95	2.98 ± 3.37	<0.001^*∗∗*^
Anxiety		113 (19.82%)	56 (15.01%)	57 (28.93%)	<0.001^*∗∗*^
PHQ-9 score		2.21 ± 3.41	1.84 ± 3.21	2.92 ± 3.66	<0.001^*∗∗*^
Depression		107 (18.77%)	57 (15.28%)	50 (25.38%)	0.003^*∗∗*^
PCS score		5.04 ± 7.91	4.51 ± 7.94	6.05 ± 7.79	<0.001^*∗∗*^
High pain catastrophizing		118 (20.70%)	65 (17.43%)	53 (26.90%)	0.008^*∗∗*^

Quantitative data presented by mean ± SD; categorial data presented by frequency (constituent ratio); ^*∗∗*^*P* < 0.01; ^*∗*^*P* < 0.05.

**Table 2:** Prevalence of TMDs subtypes and symptoms in nonanxiety and anxiety groups.

	Nonanxiety (*N* = 457)	Anxiety (*N* = 113)	Pearson *χ*^2^	*P* value
TMDs	140 (30.63%)	57 (50.44%)	15.717	<0.001^*∗∗*^
Pain-related TMDs	50 (10.94%)	20 (17.70%)	3.841	0.050
(1) TMD/facial pain	36 (7.88%)	16 (14.16%)	4.312	0.038^*∗*^
(2) Headaches	27 (5.91%)	11 (9.73%)	2.132	0.144
Intra-articular TMDs	119 (26.04%)	52 (46.02%)	17.219	<0.001^*∗∗*^
(3) TMJ noises	87 (19.04%)	36 (31.86%)	8.801	0.003^*∗∗*^
(4) Closed locking	43 (9.41%)	20 (17.70%)	6.333	0.018^*∗*^
(5) Open locking	46 (10.07%)	27 (23.89%)	15.514	<0.001^*∗∗*^

Categorial data presented by frequency (constituent ratio); ^*∗∗*^*P* < 0.01; ^*∗*^*P* < 0.05.

**Table 3:** Prevalence of TMDs subtypes and symptoms in nondepression and depression groups.

	Nondepression (*N* = 463)	Depression (*N* = 107)	Pearson *χ*^2^	*P* value
TMDs	147 (31.75%)	50 (46.73%)	8.623	0.003^*∗∗*^
Pain-related TMDs	48 (10.37%)	22 (20.56%)	8.383	0.004^*∗∗*^
(1) TMD/facial pain	36 (7.78%)	16 (14.95%)	5.401	0.020^*∗*^
(2) Headaches	25 (5.40%)	13 (12.15%)	6.364	0.012^*∗*^
Intra-articular TMDs	127 (27.43%)	44 (41.12%)	7.759	0.005^*∗∗*^
(3) TMJ noises	89 (19.22%)	34 (31.78%)	8.094	0.004^*∗∗*^
(4) Closed locking	45 (9.72%)	18 (16.82%)	4.461	0.035^*∗*^
(5) Open locking	53 (11.45%)	20 (18.69%)	4.085	0.043^*∗*^

Categorial data presented by frequency (constituent ratio); ^*∗∗*^*P* < 0.01; ^*∗*^*P* < 0.05.

**Table 4:** Prevalence of TMDs subtypes and symptoms in low and high pain catastrophizing groups.

	Low pain catastrophizing (*N* = 452)	High pain catastrophizing (*N* = 118)	Pearson *χ*^2^	*P* value
TMDs	144 (31.86%)	53 (44.92%)	7.053	0.008^*∗∗*^
Pain-related TMDs	49 (10.84%)	21 (17.80%)	4.203	0.040^*∗*^
(1) TMD/facial pain	37 (8.19%)	15 (12.71%)	2.312	0.128
(2) Headaches	24 (5.31%)	14 (11.86%)	6.461	0.011^*∗*^
Intra-articular TMDs	126 (27.88%)	45 (38.14%)	4.690	0.030^*∗*^
(3) TMJ noises	89 (19.69%)	34 (28.81%)	4.602	0.032^*∗*^
(4) Closed locking	50 (11.06%)	13 (11.02%)	0.000	0.989
(5) Open locking	51 (11.28%)	22 (18.64%)	4.540	0.033^*∗*^

Categorial data presented by frequency (constituent ratio); ^*∗∗*^*P* < 0.01; ^*∗*^*P* < 0.05.

**Table 5:** Spearman's correlations among TMDs subtypes and scores of psychological assessments.

	Intra-articular TMDs	GAD-7	PHQ-9	PCS
Pain-related TMDs	0.268^*∗∗*^	0.121^*∗∗*^	0.098^*∗*^	0.118^*∗∗*^
Intra-articular TMDs		0.171^*∗∗*^	0.157^*∗∗*^	0.066
GAD-7			0.762^*∗∗*^	0.568^*∗∗*^
PHQ-9				0.544^*∗∗*^

^*∗∗*^ *P* < 0.01 and ^*∗*^*P* < 0.05.

**Table 6:** Univariate Regression Analysis for TMDs, pain-related TMDs, and intra-articular TMDs.

Variable	*β*	OR	95% CI	*P* value
*TMDs*				
Anxiety	0.835	2.305	(1.516, 3.504)	<0.001^*∗∗*^
Depression	0.634	1.886	(1.230, 2.891)	0.004^*∗∗*^
High pain catastrophizing	0.556	1.744	(1.154, 2.636)	0.008^*∗∗*^

*Pain-related TMDs*				
Anxiety	0.560	1.751	(0.995, 3.081)	0.052
Depression	0.805	2.238	(1.283, 3.902)	0.005^*∗∗*^
High pain catastrophizing	0.577	1.781	(1.020, 3.108)	0.042^*∗*^

*Intra-articular TMDs*				
Anxiety	0.884	2.421	(1.583, 3.703)	<0.001^*∗∗*^
Depression	0.614	1.848	(1.195, 2.857)	0.006^*∗∗*^
High pain catastrophizing	0.467	1.595	(1.043, 2.439)	0.031^*∗*^

^*∗∗*^ *P* < 0.01 and ^*∗*^*P* < 0.05.

**Table 7:** Adjusted Regression Model for TMDs, pain-related TMDs, and intra-articular TMDs.

Variable	*β*	OR	95% CI	*P* value
*TMDs*				
Anxiety	0.786	2.196	(1.436, 3.358)	<0.001^*∗∗*^
Depression	0.554	1.741	(1.129, 2.685)	0.012^*∗*^
High pain catastrophizing	0.471	1.601	(1.052, 2.436)	0.028^*∗*^

*Pain-related TMDs*				
Anxiety	0.498	1.645	(0.930, 2.912)	0.087
Depression	0.795	2.136	(1.214, 3.756)	0.008^*∗∗*^
High pain catastrophizing	0.522	1.685	(0.957, 2.967)	0.071

*Intra-articular TMDs*				
Anxiety	0.850	2.341	(1.522, 3.600)	<0.001^*∗∗*^
Depression	0.546	1.726	(1.110, 2.684)	0.015^*∗*^
High pain catastrophizing	0.388	1.473	(0.958, 2.267)	0.078

^*∗∗*^ *P* < 0.01 and ^*∗*^*P* < 0.05.

## Data Availability

The data used to support the findings of this study are available from the corresponding author upon reasonable request.
